# Survey of *n-*3 and *n*-6 polyunsaturated fatty acids in fish and fish products

**DOI:** 10.1186/1476-511X-11-144

**Published:** 2012-10-30

**Authors:** Claudia Strobel, Gerhard Jahreis, Katrin Kuhnt

**Affiliations:** 1 Department of Nutritional Physiology, Institute of Nutrition, Friedrich Schiller University Jena, Dornburger Str. 24, Jena, Germany

**Keywords:** Fish, Aquatic food products, *n*-3 PUFA, *n*-6 PUFA, Aquaculture, Wild salmon, EPA, DHA, Freshwater, Marine

## Abstract

**Background:**

The imbalance of the *n*-3/*n*-6 ratio in the Western diet is characterised by a low intake of *n*-3 long-chain (LC) PUFA and a concurrent high intake of *n*-6 PUFA. Fish, in particular marine fish, is a unique source of *n*-3 LC PUFA. However, FA composition of consumed fish changed, due to the increasing usage of *n*-6 PUFA-rich vegetable oils in aquaculture feed and in fish processing (frying) which both lead to a further shift in *n*-6 PUFA to the detriment of *n*-3 LC PUFA.

The aim of this study was to determine the ratio of *n*-3/*n*-6 including the contents of EPA and DHA in fish fillets and fish products from the German market (n=123). Furthermore, the study focussed on the FA content in farmed salmon compared to wild salmon as well as in processed Alaska pollock fillet, e.g., fish fingers.

**Results:**

Total fat and FA content in fish products varied considerably depending on fish species, feed management, and food processing. Mackerel, herring and trout fillets characteristically contained adequate dietary amounts of absolute EPA and DHA, due to their high fat contents. However, despite a lower fat content, tuna, pollock, and Alaska pollock can contribute considerable amounts of EPA and DHA to the human supply.

Farmed salmon are an appropriate source of EPA and DHA owing to their higher fat content compared to wild salmon (12.3 vs. 2.1 wt %), however with elevated SFA, *n*-9 and *n*-6 FA contents representing the use of vegetable oils and oilseeds in aquaculture feed. The *n*-3/*n*-6 ratio was deteriorated (2.9 vs. 12.4) but still acceptable. Compared to pure fish fillets, breaded and pre-fried Alaska pollock fillet contained extraordinarily high fat and *n*-6 PUFA levels.

**Conclusions:**

Since fish species vary with respect to their *n*-3 LC PUFA contents, eating a variety of fish is advisable. High *n*-6 PUFA containing pre-fried fish support the imbalance of *n*-3/*n*-6 ratio in the Western diet. Thus, consumption of pure fish fillets is to be favoured. The lower *n*-3 PUFA portion in farmed fish can be offset by the higher fat content, however, with an unfavourable FA distribution compared to wild fellows.

## Background

Fish and shellfish are considered to be nutritionally high-quality food as they are a major source of marine-derived omega-3 long-chain polyunsaturated fatty acids (*n*-3 LC PUFA; C ≥ 20) for the human diet [1,2]. In humans, *n*-3 LC PUFA together with their metabolites (eicosanoids) engaged in various physiological processes and are essential for normal growth and development [3]. They also play an important role in the prevention of cardiovascular and inflammatory diseases and have a promising impact on prevention of cognitive decline and dementia in older people [3-7].

Since *n*-3 and *n*-6 PUFA compete for the same enzymes for desaturation and elongation and each class of PUFA has a different effect on human health, an appropriate ratio of both is crucial. In fact, an imbalance is associated with the prevalence of various chronic diseases [8]. Past studies indicate that human beings evolved on a diet with a *n*-6/*n*-3 ratio of approximately 1:1 [9] and according to some authors, a ratio of between 1:1 and 5:1 is supposedly beneficial to health [8,10,11]. The current Western diet, involving a high consumption of meat and sausage products as well as an increased intake of vegetable oils (via excessive use in food processing), characterises high quantities of *n*-6 PUFA and a deficit in *n*-3 PUFA [12,13]. Vegetable oils contain high quantities of *n*-6 PUFA mainly as linoleic acid (C18:2 *n*-6; LA). The increase in consumption of LA-rich oils (e.g., soybean oil, sunflower oil) is even associated with a decreased *n*-3 LC PUFA content in human body tissue [12]. Current data reveals the *n*-6/*n*-3 ratio in the Western diet as being between 8:1 and 17:1 [9,14]. Hence, it follows that by decreasing *n*-6 PUFA and simultaneously increasing *n*-3 PUFA intake this ratio can be improved leading to positive effects for human health [8,11].

Increased fish consumption, especially of marine origin, can lead to a raised supply of *n*-3 LC PUFA. For this reason, dieticians advocate eating fish at least twice a week, with preferably one meal consisting of oily marine fish such as herring, mackerel or salmon [15]. However, the recommended intake for the marine-derived LC PUFA eicosapentaenoic acid (C20:5 *n*-3; EPA) and docosahexaenoic acid (C22:6 *n*-3; DHA) as well as for total intake of *n*-3 PUFA varies between the different scientific panels. Recommendations for total EPA and DHA are between 200 mg/d and 500 mg/d [16,17]. The estimated current intake in the European population varies between 80 mg/d to 420 mg/d [16].

Since fish and mammals lack Δ12 and Δ15 desaturases responsible for converting oleic acid (18:1 *n*-9; OA) into LA and α-linolenic acid (18:3 *n*-3; ALA), they are unable to synthesize *n*-3 LC PUFA *de novo* from precursors and a dietary intake of LA and ALA is essential [18,19]. In both fish and humans, *n*-3 LC PUFA supply principally depends on the diet and on the ability to elongate and desaturate plant-derived ALA to their longer C_20_ and C_22_ homologues such as EPA and DHA [20,21].

The basic FA composition which is cumulative in the marine food chain depends on the primary producers such as marine phytoplankton (microalgae like diatome) [18,22]. These primary marine producers can effectively synthesize LC PUFA from ALA and LA via a series of desaturation and elongation reactions. Finally, they are able to directly synthesis DHA from docosapentaenoic acid (C22:5 *n*-3; DPA) via Δ4 desaturation [22-24]. Interestingly, some microbes use a polyketide synthase-like reaction to produce PUFA [22,24,25]. Whereas the marine food chain is rich in EPA and DHA, the freshwater food system contains higher levels of LA and ALA [19,26] leading to obvious differences between freshwater and marine fish in terms of FA distribution. However, some freshwater fish species can also serve as a valuable source of EPA and DHA [27-29], which they derive from freshwater plankton [30] and by endogenous metabolism [19].

Due to the high global demands for fish and the resulting overfishing of the seas (‘fishing down marine/or aquatic food webs’ [31-33], marine and freshwater aquaculture has taken on increasing importance. For example, farming and consumption of salmonids has dramatically increased during the past 20 years [33]. Furthermore, some species of freshwater fish are becoming increasingly popular amongst consumers and are heavily cultivated. One example is the iridescent shark (*Pangasius hypophthalmus*). It was one of the most frequently consumed fish species in Germany in 2009 [34].

The diet of wild fish is very different from that of their fellow species in aquaculture. In fact, aquaculture of finfish and crustaceans is still highly dependent upon marine capture fisheries that provide key dietary nutrients, such as fish meal and fish oil [35]. Fish oil is the primary source of *n*-3 LC PUFA in aquaculture [33,36,37]. Since, marine fish oils comprise a limiting factor for the strongly growing fish farming industry (5-10% per annum; [38]) aquaculture diets contain a wide variety of alternative plant-based ingredients such as legume seeds, oilseed cakes, leaf meals and an increasing portion of vegetable oils [36,39]. However, replacing fish oils with vegetable oils means that less *n*-3 LC PUFA are available in the fish diet, although in some oils such as flaxseed oil, a quantity of ALA is present and can be converted only to a limited extent into LC metabolites in fish. In general, vegetable oils in fish feed can have a detrimental effect on the FA distribution in fish as they can alter the *n*-3/*n*-6 ratio [18].

In view of the fact that demand for fish is globally increasing and that fish consumption is also generally recommended by dieticians, surprisingly, current overview of FA profiles and FA content in different fish species and in food products derived from marine and freshwater fish is lacking in the literature. Therefore, the aim of this work was to analyse FA distribution (particularly *n*-3 and *n*-6 PUFA) in various fish products and in fillets of frequently consumed fish species available on the German market. Moreover, a focus was additionally placed on analysing the FA content in farmed compared to wild salmon as well as in processed Alaska pollock fillet (e.g. fish fingers).

## Results

### Total fat and fatty acids in various fish products and fish species

Total fat content of fresh weight of all analysed samples varied between 0.39% (monkfish fillet) and 40.4% (cod liver; Table 1). The total *n*-3 PUFA proportion ranged from 2.96 ± 0.61% of FAME (iridescent shark) to 51.2 ± 3.69% of FAME (cod fillet) (Table 1). The largest fraction of *n*-3 PUFA constituted DHA, EPA and ALA, while DHA was generally higher in contrast to EPA (Table 2). Comparing the FA proportions of the seven most frequently consumed fish fillet types in Germany showed that pollock fillets contained the highest portion of EPA and DHA (44.3% of FAME), whereas iridescent shark once more contained the lowest levels (1.66% of FAME; Table 2). The total fat content is particularly important for FA concentration with respect to the fresh weight. Iridescent shark also had the least absolute *n*-3 PUFA content (0.04 ± 0.02 wt %). Fillets of herring, rainbow trout, and salmon (average of wild and farmed) showed the highest absolute contents of EPA and DHA related to high fat content (Table 2).

**Table 1:** **Total fat, *n *-3 and *n *-6 PUFA contents of different fish species**

	**Total fat**	**∑ EPA & DHA**		**∑ *n*-3 PUFA**		**∑ *n*-6 PUFA**	
**Fish species**	**[wt %]**	**[% of FAME]**	**[wt %]**	**[% of FAME]**	**[wt %]**	**[% of FAME]**	**[wt %]**
**(alphabetical order)**	**Mean ± SD**	**Mean ± SD**	**Mean ± SD**	**Mean ± SD**	**Mean ± SD**	**Mean ± SD**	**Mean ± SD**
Alaska pollock^1^ (n=17)	3.71 ± 3.71	22.8 ± 19.0	0.22 ± 0.10	25.0 ± 19.4	0.29 ± 0.10	28.6 ± 21.6	1.64 ± 1.96
Anchovies^2^ (n=1)	6.21	11.1	0.66	15.8	0.93	39.2	2.31
Black halibut, smoked (n=1)	20.4	9.39	1.82	13.0	2.52	2.06	0.40
Butterfish, smoked (n=1)	23.7	3.61	0.81	5.46	1.23	3.33	0.75
Catfish, canned fish (n=1)	2.29	0.86	0.02	4.62	0.10	15.8	0.34
Cod fillet (n=3)	0.56 ± 0.14	48.8 ± 3.44	0.26 ± 0.08	51.2 ± 3.69	0.28 ± 0.08	4.34 ± 2.21	0.02 ± 0.02
Cod liver (n=1)	40.4	18.1	6.94	22.0	8.45	2.51	0.96
Cod, smoked (n=1)	3.39	14.6	0.47	16.5	0.53	3.37	0.11
Eel, smoked (n=1)	34.9	5.01	1.66	6.78	2.25	3.39	1.12
Fried calamari^3^ (n=2)	9.45 ± 1.83	2.32 ± 0.58	0.20 ± 0.01	5.57 ± 3.77	0.53 ± 0.44	39.4 ± 30.5	3.27 ± 2.05
Herring (n=9)	12.7 ± 3.09	12.0 ± 2.10	1.45 ± 0.42	16.1 ± 2.69	1.95 ± 0.60	2.58 ± 0.41	0.31 ± 0.10
Iridescent shark (n=10)	1.54 ± 0.49	1.66 ± 0.45	0.02 ± 0.01	2.96 ± 0.61	0.04 ± 0.02	12.2 ± 2.16	0.18 ± 0.06
Louisiana-crayfish (n=1)	0.85	14.2	0.11	22.8	0.19	18.0	0.15
Mackerel (n=3)	20.6 ± 7.57	15.8 ± 0.99	3.08 ± 1.08	23.8 ± 1.48	4.64 ± 1.65	2.94 ± 0.25	0.59 ± 0.26
Monkfish (n=1)	0.39	33.7	0.12	36.0	0.13	8.25	0.03
New Zealand hake (n=1)	1.55	16.4	0.24	19.9	0.29	3.01	0.04
Plaice (n=1)	1.64	24.6	0.38	33.9	0.53	3.55	0.06
Pollock (n=10)	0.99 ± 0.37	44.3 ± 5.63	0.41 ± 0.15	46.8 ± 5.65	0.43 ± 0.15	4.27 ± 2.37	0.04 ± 0.02
Prawn (n=4)	0.53 ± 0.19	20.0 ± 1.80	0.10 ± 0.04	22.6 ± 1.19	0.11 ± 0.04	17.1 ± 4.10	0.09 ± 0.04
Redfish, fillet (n=1)	1.49	21.5	0.30	23.5	0.33	3.05	0.04
Redfish, smoked (n=1)	8.27	15.6	1.23	19.3	1.52	2.86	0.22
Salmon; farmed (n=11)	12.3 ± 4.72	13.7 ± 3.92	1.53 ± 0.52	21.2 ± 4.03	2.40 ± 0.78	10.6 ± 6.23	1.32 ± 1.07
Salmon; wild (n=10)	2.07 ± 1.14	24.2 ± 7.99	0.44 ± 0.17	28.9 ± 8.97	0.53 ± 0.21	2.35 ± 0.43	0.05 ± 0.03
Sardine^4^ (n=2)	10.2 ± 1.81	12.0 ± 0.83	1.16 ± 0.13	14.5 ± 0.75	1.40 ± 0.18	23.8 ± 17.8	2.45 ± 2.13
Spined loach, fillet (n=1)	0.74	28.7	0.20	32.6	0.23	4.55	0.03
Spiny dogfish^5^ (n=2)	33.8 ± 1.95	18.8 ± 0.61	6.03 ± 0.54	23.1 ± 0.23	7.41 ± 0.50	5.94 ± 2.09	1.89 ± 0.56
Sprat, smoked (n=2)	17.9 ± 3.01	12.0 ± 0.22	2.03 ± 0.30	16.1 ± 1.32	2.75 ± 0.68	4.06 ± 2.77	0.73 ± 0.59
Tilapia (n=1)	1.63	3.99	0.06	7.89	0.12	18.6	0.29
Trout (n=10)	7.93 ± 3.79	16.6 ± 4.44	1.17 ± 0.48	21.9 ± 4.38	1.58 ± 0.69	12.2 ± 4.11	0.94 ± 0.60
Tuna (n=10)	2.28 ± 2.59	32.9 ± 5.18	0.72 ± 0.83	35.1 ± 4.84	0.78 ± 0.92	7.67 ± 1.70	0.14 ± 0.08
Victoria perch, fillet (n=1)	0.80	10.4	0.08	16.2	0.12	5.91	0.05
Whitefish, smoked (n=1)	4.12	18.7	0.73	27.6	1.08	7.72	0.30
Zander fillet (n=1)	0.50	31.4	0.15	36.6	0.17	13.2	0.06

^1^ including fish fingers with coating, pure fillets and fillets with removed coating, ^2^ in a tin with oil (drained), ^3^in a crispy coating, ^4^ in a tin with oil (drained), ^5^curled strips of smoked dogfish; wt %, % of fresh weight.

**Table 2:** Total fat content and FA proportion of fillets of the seven most frequently consumed fish species

	**Marine**				**Freshwater/marine**	**Freshwater (Aquaculture)**	
	**Herring**^**1**^	**Tuna**^**2**^	**Pollock**^**3**^	**Alaska pollock**^**4**^	**Salmon**^**5**^	**Rainbow trout**^**6**^	**Iridescent shark**^**7**^
	**(n = 9)**	**(n = 10)**	**(n = 10)**	**(n = 10)**	**(n = 21)**	**(n = 10)**	**(n = 10)**
	**Mean ± SD**	**Mean ± SD**	**Mean ± SD**	**Mean ± SD**	**Mean ± SD**	**Mean ± SD**	**Mean ± SD**
**Total fat** [wt %]	**12.7 ± 3.09**^**a**^	**2.28 ± 2.59**^**c**^	**0.99 ± 0.37**^**c**^	**0.81 ± 0.27**^**c**^	**7.43 ± 6.25**^**b**^	**7.93 ± 3.79**^**b**^	**1.54 ± 0.49**^**c**^
**FA** [% of FAME]							
C16:0	13.3 ± 1.35^f^	22.3 ± 2.79^b^	18.9 ± 1.72^c^	17.7 ± 2.48^cd^	15.0 ± 2.98^ef^	16.0 ± 1.64^de^	28.9 ± 2.00^a^
C18:0	1.07 ± 0.12^d^	8.15 ± 1.12^a^	4.66 ± 0.85^b^	3.09 ± 0.35^c^	2.92 ± 0.56^c^	3.42 ± 0.53^c^	8.20 ± 0.76^a^
C18:1 *n*-9 (OA)	10.1 ± 3.86^d^	11.5 ± 1.93^d^	9.67 ± 2.81^d^	13.1 ± 4.78^d^	18.1 ± 7.65^c^	23.9 ± 3.82^b^	37.4 ± 1.03^a^
**∑ *n*-3 PUFA**	**16.1 ± 2.69**^**d**^	**35.1 ± 4.84**^**b**^	**46.8 ± 5.65**^**a**^	**39.8 ± 8.65**^**b**^	**24.9 ± 7.74**^**c**^	**21.9 ± 4.38**^**c**^	**2.96 ± 0.61**^**e**^
C18:3 *n*-3 (ALA)	1.08 ± 0.16^bc^	0.29 ± 0.13^c^	0.43 ± 0.10^c^	0.73 ± 0.54^c^	1.47 ± 1.20^ab^	1.91 ± 0.57^a^	0.54 ± 0.42^c^
C18:4 *n*-3 (SDA)	1.93 ± 0.64^a^	0.38 ± 0.32^de^	0.49 ± 0.19^cd^	0.35 ± 0.10^de^	1.04 ± 0.40^b^	0.78 ± 0.34^bc^	0.04 ± 0.02^e^
C20:4 *n*-3	0.42 ± 0.12^c^	0.26 ± 0.16^c^	0.45 ± 0.11^c^	0.28 ± 0.08^c^	0.99 ± 0.35^a^	0.81 ± 0.17^b^	0.05 ± 0.01^d^
C20:5 *n*-3 (EPA)	5.21 ± 1.10^cd^	4.42 ± 1.37^d^	9.82 ± 1.66^b^	14.5 ± 3.37^a^	6.45 ± 2.18^c^	4.07 ± 1.31^d^	0.22 ± 0.05^e^
C22:5 *n*-3 (DPA)	0.53 ± 0.09^cd^	0.87 ± 0.41^cd^	0.91 ± 0.25^cd^	1.04 ± 0.29^c^	2.38 ± 0.89^a^	1.56 ± 0.44^b^	0.31 ± 0.04^d^
C22:6 *n*-3 (DHA)	6.83 ± 1.44^e^	28.4 ± 4.92^b^	34.5 ± 4.48^a^	22.8 ± 5.27^c^	12.3 ± 6.44^d^	12.5 ± 3.58^d^	1.44 ± 0.41^f^
∑ EPA & DHA	12.0 ± 2.10^d^	32.9 ± 5.18^b^	44.3 ± 5.63^a^	37.3 ± 8.57^b^	18.7 ± 8.08^c^	16.6 ± 4.44^cd^	1.66 ± 0.45^e^
**∑ *n*-6 PUFA**	**2.58 ± 0.41**^**b**^	**7.67 ± 1.70**^**ab**^	**4.27 ± 2.37**^**b**^	**12.6 ± 9.71**^**a**^	**6.65 ± 6.09**^**ab**^	**12.2 ± 4.11**^**a**^	**12.2 ± 2.16**^**a**^
C18:2 *n*-6 (LA)	1.90 ± 0.50^c^	1.25 ± 0.25^c^	2.15 ± 2.51^c^	11.4 ± 9.94^a^	5.24 ± 5.77^bc^	10.3 ± 3.97^ab^	8.15 ± 1.88^ab^
C18:3 *n*-6 (GLA)	0.11 ± 0.03^b^	0.14 ± 0.04^b^	0.09 ± 0.02^b^	0.11 ± 0.03^b^	0.23 ± 0.12^a^	0.27 ± 0.03^a^	0.21 ± 0.05^a^
C20:4 *n*-6 (AA)	0.21 ± 0.03^d^	3.38 ± 1.10^a^	1.32 ± 0.20^b^	0.73 ± 0.17^c^	0.40 ± 0.12^cd^	0.49 ± 0.16^cd^	1.56 ± 0.45^b^
C22:5 *n*-6	0.12 ± 0.03^c^	2.50 ± 0.81^a^	0.41 ± 0.07^c^	0.16 ± 0.04^c^	0.16 ± 0.04^c^	0.22 ± 0.11^c^	0.82 ± 0.24^b^
**∑ *n*-3/∑ *n*-6**	**6.42 ± 1.70**^**bc**^	**4.97 ± 2.17**^**bc**^	**12.6 ± 3.96**^**a**^	**8.31 ± 8.53**^**b**^	**7.41 ± 5.68**^**b**^	**2.06 ± 0.91**^**c**^	**0.24 ± 0.03**^**d**^
**∑ *n*-6/∑ *n*-3**	**0.17 ± 0.04**^**c**^	**0.22 ± 0.06**^**c**^	**0.09 ± 0.06**^**c**^	**0.38 ± 0.35**^**bc**^	**0.34 ± 0.39**^**bc**^	**0.62 ± 0.39**^**b**^	**4.19 ± 0.58**^**a**^
∑ SFA	23.2 ± 2.30^c^	34.8 ± 4.00^b^	25.9 ± 2.08^c^	22.7 ± 2.67^c^	23.2 ± 3.87^c^	24.2 ± 2.32^c^	41.3 ± 2.81^a^
∑ MUFA	53.4 ± 3.88^a^	17.9 ± 4.21^c^	20.7 ± 4.77^c^	22.9 ± 3.80^c^	41.0 ± 7.19^b^	37.9 ± 3.89^b^	40.9 ± 1.13 ^b^
∑ PUFA	20.0 ± 2.65^d^	43.2 ± 5.06^b^	51.3 ± 5.62^a^	52.7 ± 2.60^a^	32.8 ± 6.92^c^	35.2 ± 2.07^c^	15.3 ± 2.66^e^
**FA** [wt %]							
**∑ *n*-3 PUFA**	**1.95 ± 0.60**^**a**^	**0.78 ± 0.92**^**b**^	**0.43 ± 0.15**^**b**^	**0.30 ± 0.11**^**b**^	**1.51 ± 1.11**^**a**^	**1.58 ± 0.69**^**a**^	**0.04 ± 0.02**^**b**^
C18:3 *n*-3 (ALA)	0.13 ± 0.04^ab^	0.01 ± 0.01^b^	≤0.01 ± 0.00^b^	0.01 ± 0.01^b^	0.15 ± 0.20^a^	0.15 ± 0.10^a^	0.01 ± 0.01^b^
C20:5 *n*-3 (EPA)	0.64 ± 0.23^a^	0.12 ± 0.20^cd^	0.09 ± 0.04^cd^	0.11 ± 0.04^cd^	0.41 ± 0.32^b^	0.30 ± 0.20^bc^	≤0.01 ± 0.00^d^
C22:6 *n*-3 (DHA)	0.81 ± 0.21^a^	0.60 ± 0.63^ab^	0.32 ± 0.11^bc^	0.17 ± 0.06^c^	0.61 ± 0.37^ab^	0.86 ± 0.30^a^	0.02 ± 0.01^c^
**∑ *n*-6 PUFA**	**0.31 ± 0.10**^**b**^	**0.14 ± 0.08**^**b**^	**0.04 ± 0.02**^**b**^	**0.10 ± 0.10**^**b**^	**0.71 ± 1.00**^**ab**^	**0.94 ± 0.60**^**a**^	**0.18 ± 0.06**^**b**^
C18:2 *n*-6 (LA)	0.23 ± 0.09^b^	0.03 ± 0.04^b^	0.02 ± 0.02^b^	0.10 ± 0.10^b^	0.59 ± 0.90^ab^	0.80 ± 0.55^a^	0.12 ± 0.04^b^
C20:4 *n*-6 (AA)	0.03 ± 0.01^bcd^	0.05 ± 0.03^a^	0.01 ± 0.01^cd^	0.01 ± 0.00^d^	0.03 ± 0.02^bc^	0.04 ± 0.02^b^	0.02 ± 0.01^bcd^

^1^ contains herring fillets (*Clupea harengus*) in a tin (drained); ^2^ consists of *Thunnus albacares*, *Thunnus Alalunga*, *Katsuwonus pelamis*; ^3^*Pollachius virens*; ^4^*Theragra chalcogramma*, consists of pure fillets and fillets with removed coating; ^5^ consists of wild salmon (*Oncorhynchus keta*, *Oncorhynchus gorbuscha*, *Oncorhynchus nerka*) and salmon from aquaculture (*Salmo salar*); ^6^*Oncorhynchus mykiss*; ^7^*Pangasius hypophthalamus*.^abc^ Means with different superscript letters indicate significant differences, *P* ≤ 0.05. *AA*, arachidonic acid; *ALA*, α-linolenic acid; *DHA*, docosahexaenoic acid; *DPA*, docosapentaenoic acid; *EPA*, eicosapentaenoic acid; *GLA*, γ-linolenic acid; *LA*, linoleic acid; *OA*, oleic acid; *SDA*, stearidonic acid.

### Comparison of wild and farmed salmon

Fillets from wild and farmed salmon differed with regard to fat content and FA distribution (Table 3). Fat content in fillets from cultured salmon was significantly higher than from the wild counterpart (12.3 vs. 2.07 wt %). In terms of FAME, wild salmon showed a significantly higher proportion of total *n*-3 PUFA (28.9% vs. 21.2%), which resulted mainly from higher EPA (7.29%) and DHA (16.9% of FAME; Table 3). In contrast, cultured salmon exhibited greater absolute amounts of total *n*-3 PUFA, which were, however, associated with a higher fat intake consisting mainly of SFA, OA and LA (Table 3).

**Table 3:** Total fat and FA proportion of wild and farmed salmon

	**Salmon fillet**		**Salmon fillet**	
	**Relative FA amount [% of FAME]**		**Absolute FA amount [% of fresh weight]**	
	**Wild**^**1**^	**Farmed**^**2**^	**Wild**	**Farmed**
	**(n = 10)**	**(n = 11)**	**(n = 10)**	**(n = 11)**
	**Mean ± SD**	**Mean ± SD *P***^**3**^	**Mean ± SD**	**Mean ± SD *P***^**4**^
**Total Fat** [wt %]			**2.07 ± 1.14**	12.3 ± 4.72*
**FA**				
C16:0	16.6 ± 3.22	13.6 ± 1.85*	0.32 ± 0.16	1.57 ± 0.60*
C18:0	2.98 ± 0.56	2.86 ± 0.59	0.05 ± 0.02	0.34 ± 0.17*
C18:1 *n*-9 (OA)	13.4 ± 2.39	22.3 ± 8.42*	0.27 ± 0.17	2.75 ± 1.83*
**∑ *n-*3 PUFA**	**28.9 ± 8.97**	21.2 ± 4.03*	**0.53 ± 0.21**	2.40 ± 0.78*
C18:3 *n*-3 (ALA)	0.58 ± 0.19	2.29 ± 1.15*	0.01 ± 0.01	0.28 ± 0.20*
C18:4 *n*-3 (SDA)	0.94 ± 0.36	1.13 ± 0.43	0.02 ± 0.01	0.12 ± 0.05*
C20:4 *n*-3	0.80 ± 0.23	1.17 ± 0.35*	0.02 ± 0.01	0.13 ± 0.05*
C20:5 *n*-3 (EPA)	7.29 ± 2.29	5.69 ± 1.85	0.14 ± 0.07	0.65 ± 0.26*
C22:5 *n*-3 (DPA)	2.21 ± 1.04	2.53 ± 0.74	0.04 ± 0.02	0.30 ± 0.12*
C22:6 *n*-3 (DHA)	16.9 ± 6.18	8.04 ± 2.67*	0.31 ± 0.11	0.88 ± 0.29*
∑ EPA & DHA	24.2 ± 7.99	13.7 ± 3.92*	0.44 ± 0.17	1.53 ± 0.52*
**∑ *n*-6 PUFA**	**2.35 ± 0.43**	10.6 ± 6.23*	**0.05 ± 0.03**	1.32 ± 1.07*
C18:2 *n*-6 (LA)	1.27 ± 0.32	8.85 ± 6.04*	0.02 ± 0.01	1.11 ± 1.00*
C18:3 *n*-6 (GLA)	0.14 ± 0.05	0.30 ± 0.10*	≤0.01 ± 0.00	0.04 ± 0.02*
C20:4 *n*-6 (AA)	0.40 ± 0.11	0.40 ± 0.13	0.01 ± 0.00	0.05 ± 0.02*
C22:5 *n*-6	0.15 ± 0.04	0.17 ± 0.04	≤0.01 ± 0.00	0.02 ± 0.01*
**∑ *n*-3/∑ *n*-6**	**12.4 ± 3.86**	2.89 ± 1.96*	**12.4 ± 3.86**	2.89 ± 1.96*
**∑ *n*-6/∑ *n*-3**	**0.09 ± 0.03**	0.56 ± 0.42*	**0.09 ± 0.03**	0.56 ± 0.42*
∑ SFA	24.7 ± 4.51	21.9 ± 2.76	0.47 ± 0.22	2.54 ± 0.95*
∑ MUFA	39.5 ± 8.25	42.3 ± 6.17	0.83 ± 0.63	4.96 ± 2.22*
∑ PUFA	32.0 ± 9.19	33.5 ± 4.32	0.59 ± 0.24	3.92 ± 1.56*

^1^ Wild salmon (*Oncorhynchus keta, Oncorhynchus gorbuscha, Oncorhynchus nerka*); ^2^ salmon from aquaculture (*Salmo salar*), ^3^ significant differences between wild and aquaculture salmon in terms of % of FAME, *P* ≤ 0.05; ^4^ significant differences between wild and aquaculture salmon in terms of % of fresh weight, *P* ≤ 0.05. *AA*, arachidonic acid; *ALA*, α-linolenic acid; *DHA*, docosahexaenoic acid; *DPA*, docosapentaenoic acid; *EPA*, eicosapentaenoic acid; *GLA*, γ-linolenic acid; *LA*, linoleic acid; *OA*, oleic acid; *SDA*, stearidonic acid.

### Comparison of breaded and pure Alaska pollock fillet

Fish fingers contained significantly higher total fat and *n*-6 PUFA compared to pure Alaska pollock fillets (Table 4). In addition, the breaded pre-fried products had increased MUFA (mainly OA) and SFA levels in relation to fat. Although Alaska pollock fish fingers with removed breading showed significantly lower *n*-6 PUFA (19.0% of FAME) in contrast to the breaded products (51.5% of FAME), these concentrations were still higher in comparison to pure fillets (2.6% of FAME). The *n*-3 PUFA portion was the lowest in fish fingers, but with reference to the higher fat content, the absolute *n*-3 PUFA per 100 g product was analogous (0.39, 0.30, 0.30 wt %). However, when separated into single *n*-3 PUFA, it is evident that EPA and DHA in fish fingers showed significantly the lowest levels (Table 4) in accordance with the decreased fish portion per 100 g of fish fingers (Table 4).

**Table 4:** **Total fat content, FA proportion and FA content of Alaska pollock**^**1**^**products depending on breading**

	**Relative FA amount [% of FAME]**			**Absolute FA amount [% of fresh weight]**		
	**Pure fillets**	**Fish fingers with removed coating**	**Fish fingers with coating**	**Pure fillets**	**Fish fingers with removed coating**	**Fish fingers with coating**
	**(n = 4)**	**(n = 6)**	**(n = 7)**	**(n = 4)**	**(n = 6)**	**(n = 7)**
	**Mean ± SD**	**Mean ± SD**	**Mean ± SD**	**Mean ± SD**	**Mean ± SD**	**Mean ± SD**
**Total fat [wt %]**				**0.77 ± 0.31**^**b**^	**0.88 ± 0.26**^**b**^	**7.85 ± 1.61**^**a**^
**FA**						
C16:0	20.7 ± 1.55^a^	16.0 ± 0.83^b^	7.48 ± 1.75^c^	0.16 ± 0.01^b^	0.14 ± 0.01^b^	0.59 ± 0.14^a^
C18:0	3.36 ± 0.32^a^	3.02 ± 0.31^b^	3.36 ± 0.34^a^	0.03 ± 0.00^b^	0.03 ± 0.00^b^	0.26 ± 0.03^a^
C18:1 *n*-9 (OA)	6.81 ± 0.46^c^	16.5 ± 1.79^b^	29.6 ± 7.43^a^	0.05 ± 0.00^c^	0.15 ± 0.02^b^	2.32 ± 0.58^a^
**∑ *n*-3 PUFA**	**50.2 ± 1.09**^**a**^	**33.6 ± 4.13**^**b**^	**3.83 ± 1.60**^**c**^	**0.39 ± 0.01**^**a**^	**0.30 ± 0.04**^**a**^	**0.30 ± 0.13**^**a**^
C18:3 *n*-3 (ALA)	0.29 ± 0.08^c^	0.94 ± 0.59^b^	1.69 ± 1.58^a^	0.00 ± 0.00^b^	0.01 ± 0.01^b^	0.13 ± 0.12^a^
C18:4 *n*-3 (SDA)	0.43 ± 0.09^a^	0.30 ± 0.08^b^	0.02 ± 0.01^c^	0.00 ± 0.00^a^	0.00 ± 0.00^a^	0.00 ± 0.00^a^
C20:4 *n*-3	0.37 ± 0.07^a^	0.23 ± 0.05^b^	0.01 ± 0.01^c^	0.00 ± 0.00^a^	0.00 ± 0.00^a^	0.00 ± 0.00^a^
C20:5 *n*-3 (EPA)	18.4 ± 0.81^a^	12.1 ± 1.61^b^	0.82 ± 0.16^c^	0.14 ± 0.01^a^	0.11 ± 0.01^a^	0.06 ± 0.01^b^
C22:5 *n*-3 (DPA)	1.26 ± 0.13^a^	0.88 ± 0.27^b^	0.07 ± 0.01^c^	0.01 ± 0.00^a^	0.01 ± 0.00^a^	0.01 ± 0.00^a^
C22:6 *n*-3 (DHA)	29.4 ± 1.24^a^	19.1 ± 2.45^b^	1.20 ± 0.25^c^	0.23 ± 0.01^a^	0.17 ± 0.02^a†^	0.09 ± 0.02^b^
∑ EPA & DHA	47.8 ± 1.10^a^	31.2 ± 4.02^b^	2.02 ± 0.40^c^	0.37 ± 0.01^a^	0.27 ± 0.04^a†^	0.16 ± 0.03^b^
**∑ *n*-6 PUFA**	**2.58 ± 0.43**^**c**^	**19.0 ± 6.85**^**b**^	**51.5 ± 7.66**^**a**^	**0.02 ± 0.00**^**b**^	**0.17 ± 0.06**^**b**^	**4.04 ± 0.60**^**a**^
C18:2 *n*-6 (LA)	1.07 ± 0.46^c^	18.0 ± 6.90^b^	51.4 ± 7.67^a^	0.01 ± 0.00^b^	0.16 ± 0.06^b^	4.03 ± 0.60^a^
C18:3 *n*-6 (GLA)	0.13 ± 0.02^a^	0.09 ± 0.03^b^	0.01 ± 0.01^c^	0.00 ± 0.00^a^	0.00 ± 0.00^a^	0.00 ± 0.00^a^
C20:4 *n*-6 (AA)	0.96 ± 0.08^a^	0.61 ± 0.05^b^	0.04 ± 0.01^c^	0.01 ± 0.00^a^	0.01 ± 0.00^a^	0.00 ± 0.00^b^
C22:5 *n*-6	0.21 ± 0.02^a^	0.14 ± 0.02^b^	0.01 ± 0.01^c^	0.00 ± 0.00^a^	0.00 ± 0.00^a^	0.00 ± 0.00^a^
**∑ *n*-3/∑ *n*-6**	**19.8 ± 3.04**^**a**^	**2.19 ± 1.48**^**b**^	**0.08 ± 0.04**^**c**^	**19.8 ± 3.04**^**a**^	**2.19 ± 1.48**^**b**^	**0.08 ± 0.04**^**c**^
**∑ *n*-6/∑ *n*-3**	**0.05 ± 0.01**^**c**^	**0.59 ± 0.28**^**b**^	**16.0 ± 7.67**^**a**^	**0.05 ± 0.01**^**c**^	**0.59 ± 0.28**^**b**^	**16.0 ± 7.67**^**a**^
∑ SFA	26.0 ± 1.14^a^	20.7 ± 0.69^b^	12.2 ± 1.87^c^	0.20 ± 0.01^b^	0.18 ± 0.01^b^	0.96 ± 0.15^a^
∑ MUFA	18.4 ± 0.34^b^	24.9 ± 3.00^ab^	31.7 ± 7.96^a^	0.14 ± 0.00^b^	0.22 ± 0.03^b^	2.49 ± 0.62^b^
∑ PUFA	53.2 ± 1.29^a^	52.9 ± 3.11^a^	55.5 ± 6.71^a^	0.41 ± 0.01^b^	0.47 ± 0.03^b^	4.36 ± 0.53^a^

^**1**^*Theragra chalcogramma.*^abc^ Means with different superscript letters indicate significant differences, *P*≤0.05; ^†^*P*≤0.1 compared to pure fillets. *AA*, arachidonic acid; *ALA*, α-linolenic acid; *DHA*, docosahexaenoic acid; *DPA*, docosapentaenoic acid; *EPA*, eicosapentaenoic acid; *GLA*, γ-linolenic acid; *LA*, linoleic acid; *OA*, oleic acid; *SDA*, stearidonic acid.

## Discussion

### Total fat and *n*-3 PUFA in various fish products and fish fillets

The total fat content and the proportion of *n*-3 PUFA varied greatly amongst the fish species and the fish products. It is not surprising that due to their increased absolute EPA and DHA levels, oily sea fish such as mackerel and herring are considered to be of higher nutritional value (Tables 1, 2). In order to meet dietary EPA and DHA recommendations in human nutrition the American Heart Association (AHA) recommends the consumption of two servings (3 oz; 85 g) of fish (particularly oily fish) twice a week [40]. Referring to the fat content, the German Nutrition Society (DGE) recommends a lower serving of oily fish (e.g., 70 g) and a greater serving of lean fish (80–150 g) once a week [41]. This illustrates the importance of analysing the total fat and its relation to the absolute FA content in fish species. Nonetheless, according to the data obtained in this study, we can safely assume that, even the other fish species frequently consumed in Germany are adequate sources of *n*-3 LC PUFA (Table 2). In contrast, the increasingly consumed iridescent shark and tilapia fails to sufficiently contribute towards *n*-3 PUFA supply in humans (Tables 1, 2).

In general, fat and FA content in fish vary greatly both between and within fish species as values are mainly influenced by feeding as well as further factors such as the geographical location of the fishing ground, season, food availability, water temperature, age and size of the fish and the maturation status [42-47]. This can be seen in the fact that published data varies to a great extent with respect to FA content in fish. In addition, it is well known that lipid distribution in fish is not uniform and fluctuates throughout the body [44,48]. In cod, for example, reserve fat is stored in the liver [49] and not between the muscle fibrils as in oily fish, rendering cod fillet very lean (in liver the fat content is 67 times higher compared to fillet; Table 1). Therefore, despite cod fillets containing significantly higher *n*-3 PUFA (51.2 ± 3.7% of FAME) than cod liver (22.0% of FAME) but the absolute *n*-3 PUFA content was low (0.28% vs. 8.5%; Table 1).

### Differences in FA profile and FA contents between wild and farmed fish

Wild salmon is also known as Pacific salmon [44]. The relatively high *n*-3 PUFA and low *n*-6 PUFA proportion found in wild salmon is directly related to their diet. Wild salmon are predatory fish that feed on smaller fish, e.g. herring, sprat or shellfish, which contain elevated *n*-3 LC PUFA.

Salmon and trout are major cultivated species raised on compound aquaculture feeds [35]. The bulk of Atlantic salmon (*Salmo salar*) available on the market today is farmed. The feed for farmed salmon is based on fish meal and fish oil rich in *n*-3 LC PUFA [36]. Therefore, farmed fish should have similar *n*-3 PUFA levels comparable to wild fish [44]. However, because marine fish oils are a limiting factor, vegetable oils and oil seeds are frequent alternatives in aquaculture feed [35-37,50] and lead to high amounts of *n*-9 MUFA and *n*-6 PUFA as seen in farmed salmon (Table 3). Recent studies have confirmed an increase of C_18_ FA such as OA, LA, and ALA in the fillet of farmed fish through the use of vegetable oils in the feed, whilst the content of *n*-3 LC PUFA was decreased [36,51-54].

The actual noticeably higher LA and ALA found in cultured salmon and also in rainbow trout (*Oncorhynchus mykiss*; often called salmon-trout) presumably indicates the use of vegetable oils such as sunflower, soybean, rapeseed, and linseed oil in the feed (Tables 2, 3). Rainbow trout on the market originate mainly from aquaculture [35]. The relatively high EPA and DHA levels in farmed salmon and rainbow trout (13.7% and 16.6% of FAME; Tables 2, 3) can result primarily from the direct dietary intake of fish oil-containing feed [55], but also to a lesser extent from the limited endogenous conversion of ALA [19,56]. Turkey wild rainbow trout flesh contained similar amounts of total *n*-3 PUFA compared to cultured rainbow trout, however, with higher ALA and EPA and lower DHA [48].

In contrast, iridescent shark, which are also almost exclusively farmed, have only low *n*-3 PUFA level (Table 2). Industrial feed for iridescent shark (rice hulls, rice, soy flour, and bananas [57-59]) has a relatively low concentration of *n*-3 PUFA and can contain around 2-3% fish oil [57]. This has only a low impact on *n*-3 PUFA content as reflected by recent analyses. The mainly plant-based nutrition is echoed in a higher *n*-6 PUFA proportion, however, it is negligible with respect to the low fat content in fresh weight (Table 2).

The high levels of fat in the feed for farmed salmon are intended to achieve a stronger growth as well as a faster lipid deposition within a short period of time. In most cultured fish, the often lower *n*-3 PUFA portion is offset by a higher fat content [44,60]. Therefore, the absolute EPA and DHA intake when consuming 100 g of farmed salmon would be 3–4 times higher compared to eating wild salmon (Table 3), thus, identifying farmed salmon as a suitable source of *n*-3 LC PUFA. Although farmed salmon has an adequate *n*-3/*n*-6 ratio (2.9), it is lower compared to wild salmon (12.4) and consumption is accompanied by a high intake of total fat (e.g., 5 times higher SFA content). According to the present results, total fat was also higher in cultured compared to wild rainbow trout [48]. Whilst the flesh contained similar amounts, the skin of the cultured fish had a higher lipid content [48].

In general, aquaculture feeding shows regional disparities and feed can contain different lipid sources and concentrations of fish oil [39]. All in all, the conditions in aquaculture feeding are extremely heterogeneous [35] and can change rapidly with a trend to further change in the future. The precise composition of the different feeds of all the various fish samples analysed was not known, in this survey, hence no direct conclusions relating to the influence of fatty acid composition between the diets of the analysed wild and farmed salmon is possible. Moreover, we analysed only samples from the German market which additionally differ with regard to species, diet (piscivorous, plankton consuming, various aquaculture feed), fishing zone and individual genetic variation.

Clearly, this survey is limited to the analysed samples and is not representative of all farmed or wild salmon. Nevertheless, the present data can provide an indication that wild salmon have generally lower fat and *n*-6 PUFA content, resulting in a favourable *n*-3/*n*-6 ratio compared to fellow farmed species. However, due to the steadily increasing world human population together with the depleting supply of marine fish due to overfishing of the seas, obtaining the dietary recommend dose of *n*-3 LC PUFA via consumption of wild fish is not a viable solution in the long term. Farmed fish can generally be regarded as an appropriate source of EPA and DHA with respect to the higher fat content which is clearly dependent on EPA and DHA contained aquaculture feed [38]. Feeding only *n*-3 PUFA-poor vegetable oils such as soybean and palm oil as lipid source will lead to a decline of EPA and DHA in farmed fish with an increase of SFA and *n*-6 PUFA. Thus, based on this trend, fish consumption in the future will lose its unique positive health impact.

Therefore, a number of promising strategies related altering aquaculture diets with respect to providing adequate alternatives to fishmeal and fish oil are being developed and implemented in order to improve the nutritional value of commercially farmed fish in the future; e.g., culture of aquatic (partly transgenic) microbes (microalgae and yeast) which synthesize EPA and/or DHA and transgenic plants (e.g., rapeseed) which produce these *n*-3 LC PUFA [18,23,24,38,61,62].

### Influence of food processing on FA contents using the example of fish fingers

The processing of fish fillets (breading and frying in vegetable oils) had an adverse influence on total fat and FA distribution, especially on increasing LA and decreasing the *n*-3/*n*-6 ratio (0.08 vs. 2.19 vs. 19.8; Table 4). Due to the lower proportion of pure fish fillet in 100 g fish finger (at least 65% fillet), the EPA and DHA concentrations were halved, whilst ALA, OA, and LA had increased due to breading and frying (Table 4). The total *n*-6 PUFA intake is certainly higher in breaded fish foods compared to farmed salmon fillet as confirmed in the present analysis (4.0 vs. 1.3 wt %). Furthermore, removing the breading greatly reduced the high fat content in fish fingers, although, due to frying oils penetrating into the fish fillet, the *n*-6 lipid portion was still higher (Table 4). Similarly, the total fat content was increased in breaded cod fillet whilst the *n*-3/*n*-6 ratio decreased [63].

In general, the total *trans*-FA (analysed by GC-column: CP-select for FAME; 200 m) were at least below 2% of FAME (data not shown).

## Conclusion

It can be concluded that the *n*-3 PUFA content of foods of aquatic origin varies strongly depending on the fish species, the fat content of fish, and the feeding conditions. Consumption of fish and shellfish does not necessarily result in a sufficient supply of EPA and DHA in humans. Therefore, consumers should consume a variety of different fish species.

The present work confirmed that intake of the frequently consumed fish species such as herring, tuna, pollock, Alaska pollock, salmon, and rainbow trout meets the dietary recommendations for total EPA and DHA of 200–500 mg/d.

Due to their generally higher fat content, even farmed fish can be an appropriate source of EPA and DHA, although these are associated with higher amounts of *n*-6 PUFA and a lower *n*-3/*n*-6 ratio compared to wild fish. One reason for this low ratio is the increasing usage of *n*-6 PUFA-rich vegetable oils as an alternative to fish oil in aquaculture feed, resulting in a decreased nutritional value of farmed fish. In general, strategies to ensure alternative *n*-3 LC PUFA sources in aquaculture feed need to be developed and realised (e.g., transgenic microalgae and plants).

Consumption of breaded pre-fried fish should generally be avoided owing to the high fat and *n*-6 PUFA content which is known to promote the shift to an unfavourable *n*-3/*n*-6 ratio, currently a widespread phenomenon of the *n*-6 PUFA-rich Western diet.

## Materials and methods

### Samples

The fresh, canned or deep-frozen packaged fish samples (n = 123; 26 fish species and shellfish) used for the study were purchased in various German discounters, supermarkets and fish counters between 2009 and 2011. For canned fish, sauces and oils were separated from the fish fillet. Fish fingers made of Alaska pollock (*Theragra chalcogramma*) were analysed both with and without breaded coating. To determine the most frequently consumed fish species, data of fish consumption from internal studies (unpublished data) and other references (e.g., [34]) were used. The group of wild salmon comprised *Oncorhynchus keta*, *Oncorhynchus gorbuscha* and *Oncorhynchus nerka* whereas the aquaculture salmon group consisted of *Salmo salar*. Data from fish samples in Tables 2 and 3 are exclusively from fillets (skin and bones had been removed).

### Sample preparation

Samples were stored at −20°C until prepared for lyophilisation. Following lyophilisation, samples were crushed, homogenised, and stored at −20°C until further analysis. After an acid hydrolysis using 4 N hydrochloric acid (Carl Roth GmbH & Co. KG, Karlsruhe, Germany), total fat content was extracted in a SOXTHERM 2000 S306 Automatic Extractor System Gerhardt (Gerhardt; Bonn, Germany) with 140 ml of petroleum ether (Carl Roth GmbH & Co. KG; extraction time: 30 min; temperature: 150°C). The amount of total fat expressed as % (g/100 g) of fresh weight (wt %) was determined gravimetrically after drying.

Lipids for FA analysis were extracted according to Folch et al. [64] (methanol/chloroform/2% sodium chloride solution; 1/2/1 v/v/v; Merck KGaA, Darmstadt, Germany). To prevent oxidation of the sensitive *n*-3 and *n*-6 PUFA during sample processing, methanol incorporating the antioxidant 2,6-di-*tert*-butyl-4-methylphenol (BHT; 0.05%; Fluka Chemie AG, Buchs, Switzerland) was employed. We had previously verified that possible BHT peaks would not disturb the gas chromatography (GC) analysis [unpublished results]. The lipid extract was stored at −20°C until further analysis. Total lipids (25 mg) were saponified with a 0.5 M methanolic sodium hydroxide solution at 100°C (10 min). The subsequent methylation of FA to FA methyl esters (FAME) was performed using methanolic boron trifluoride (BF_3_) solution (10% w/w, Supelco; Taufkirchen, Germany) at 100°C for 5 min [65]. The efficiency of methylation was proved by thin-layer chromatography.

### GC analysis

The FAME were analysed by GC (GC-17A; Shimadzu, Kyoto, Japan) using a 60 m fused-silica capillary column of medium polarity (DB 225MS: 60 m × 0.25 mm i.d. with 0.25 mm film thickness; Agilent Technologies, USA). Conditions for GC analysis and peak integration as well as the standards used have previously been described by Kuhnt et al. [66]. In total, 86 FA were identified with a minimum area of 500 counts (2% FAME in hexane, split ratio 1:100). To identify fish oil specific peaks, we used in addition to standards (No. 463, 674: NU-CHEK PREP, INC., Elysian, U.S.; BR2, BR4, ME 93: Larodan, Malmö, Sweden; Supelco® 37 Component FAME Mix: Supelco, Bellefonte, U.S. [66]) commercial menhaden oil (PUFA-3: Matreya LLC, USA). Only identified peaks were included in the summation of the peak areas. FA were expressed as FAME of total identified FAME (FA/% of total FAME). To convert FAME into absolute FA content in total fat extracts, we used the factor 0.9 to consider the portion of glycerine (FA/% of fresh weight; wt %). The FAME were grouped into saturated FA (SFA), monounsaturated FA (MUFA), PUFA, and LC PUFA (C ≥ 20). Total *n*-3 and *n*-6 PUFA were calculated without conjugated linoleic acids.

### Statistical analysis

Statistical analysis was carried out using PASW Statistics, version 18.0 (Â©2009 SPSS Statistics 18 Inc, Illinois, USA). Results were stated as means with standard deviation (SD) and *P* ≤ 0.05 indicated significant difference. The single-factor ANOVA was used for comparing the mean value between two groups, whilst the multivariate post hoc test according to Student-Newman-Keuls was employed to compare the mean values of several food groups.

## Abbreviations

AA: Arachidonic acid; ALA: α-linolenic acid; BF_3_: Boron trifluoride; BHT: 2,6-di-*tert*-butyl-4-methylphenol; DHA: Docosahexaenoic acid; DPA: Docosapentaenoic acid; EPA: Eicosapentaenoic acid; FA: Fatty acids; FAME: Fatty acid methyl esters; GC: Gas chromatography; GLA: γ-linolenic acid; LA: Linoleic acid; LC PUFA: Long chain polyunsaturated fatty acids; MUFA: Monounsaturated fatty acids; OA: Oleic acid; PUFA: Polyunsaturated fatty acids; SDA: Stearidonic acid; SFA: Saturated fatty acids.

## Competing interests

The authors declare that they have no competing interests.

## Authors’ contributions

KK and GJ designed the study. CS and KK collected the samples, performed the fatty acid analysis and the statistical analyses. CS and KK wrote the paper. All authors read and approved the final manuscript.
